# (3*R*,4*S*,5*R*)-Methyl 3,5-bis­[(*tert*-butyl­dimethyl­sil­yl)­oxy]-4-meth­oxy­cyclo­hex-1-ene­carboxyl­ate

**DOI:** 10.1107/S1600536813007551

**Published:** 2013-04-05

**Authors:** Ri Liu, Yu Shi, Chun-Xiu Xu, Yi-Liang Li

**Affiliations:** a School of Chinese Materia Medica, Tianjin University of Traditional Chinese Medicine, Tianjin 300193, People’s Republic of China; b Tianjin Key Laboratory of Molecular Design and Drug Discovery, Tianjin Institute of Pharmaceutical Research, Tianjin 300193, People’s Republic of China

## Abstract

The title compound, C_21_H_42_O_5_Si_2_, was synthesized from (3*R*,4*S*,5*R*)-methyl 3,5-bis­[(*tert*-butyl­dimethyl­sil­yl)­oxy]-4-hy­droxy­cyclo­hex-1-ene­carboxyl­ate by an esterification reaction. The cyclo­hexene ring adopts a half-chair conformation. In the crystal, mol­ecules are linked *via* C—H⋯O hydrogen bonds, forming helical chains propagating along [010].

## Related literature

The title compound is an inter­mediate in the synthesis of vandetanib {systematic name: *N*-(4-bromo-2-fluoro­phen­yl)-6-meth­oxy-7-[(1-methyl-4-piperidin­yl)meth­oxy]-4-quinazolinamine} derivatives. For vandetanib as a tyrosine kinase inhib­itor, see: Heymach (2005 ▶); Morabito *et al.* (2009 ▶); Wells *et al.* (2010 ▶); Natale *et al.* (2009 ▶).
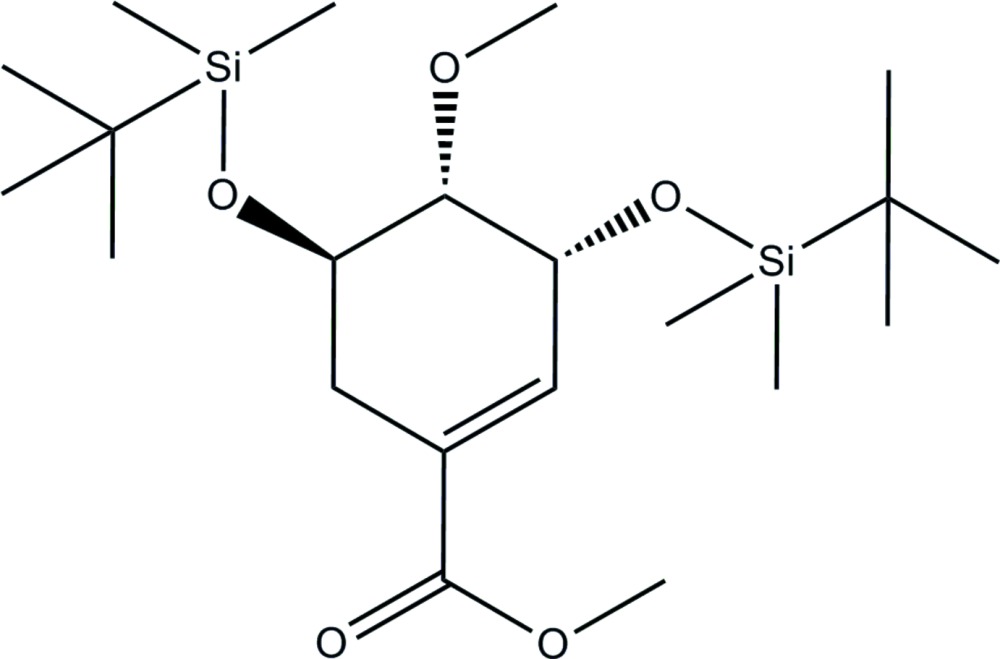


## Experimental

### 

#### Crystal data

C_21_H_42_O_5_Si_2_
*M* *_r_* = 430.72
Monoclinic, 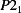

*a* = 10.760 (5) Å
*b* = 8.321 (4) Å
*c* = 14.601 (7) Å
β = 98.997 (9)°
*V* = 1291.3 (10) Å^3^
*Z* = 2
Mo *K*α radiation
μ = 0.16 mm^−1^
*T* = 113 K
0.20 × 0.18 × 0.12 mm

#### Data collection

Rigaku Saturn724 CCD diffractometer
Absorption correction: multi-scan (*CrystalClear*; Rigaku, 2007 ▶) *T*_min_ = 0.968, *T*_max_ = 0.981
13589 measured reflections
6015 independent reflections
4456 reflections with *I* > 2σ(*I*)
*R*_int_ = 0.058

#### Refinement

*R*[*F* ^2^ > 2σ(*F* ^2^)] = 0.048
*wR*(*F* ^2^) = 0.073
*S* = 0.98
6015 reflections
265 parameters
1 restraint
H-atom parameters constrained
Δρ_max_ = 0.21 e Å^−3^
Δρ_min_ = −0.29 e Å^−3^
Absolute structure: Flack (1983 ▶), 2745 Friedel pairs
Flack parameter: −0.04 (9)

### 

Data collection: *CrystalClear* (Rigaku, 2007 ▶); cell refinement: *CrystalClear*; data reduction: *CrystalClear*; program(s) used to solve structure: *SHELXS97* (Sheldrick, 2008 ▶); program(s) used to refine structure: *SHELXL97* (Sheldrick, 2008 ▶); molecular graphics: *SHELXTL* (Sheldrick, 2008 ▶); software used to prepare material for publication: *CrystalStructure* (Rigaku/MSC, 2006 ▶).

## Supplementary Material

Crystal structure: contains datablock(s) I, global. DOI: 10.1107/S1600536813007551/vm2187sup1.cif

Structure factors: contains datablock(s) I. DOI: 10.1107/S1600536813007551/vm2187Isup2.hkl

Supplementary material file. DOI: 10.1107/S1600536813007551/vm2187Isup4.cdx

Additional supplementary materials:  crystallographic information; 3D view; checkCIF report

## Figures and Tables

**Table 1:** Hydrogen-bond geometry (Å, °)

*D*—H⋯*A*	*D*—H	H⋯*A*	*D*⋯*A*	*D*—H⋯*A*
C7—H7*B*⋯O3^i^	0.98	2.55	3.410 (3)	147
C9—H9*A*⋯O3^ii^	0.98	2.59	3.527 (3)	161

Symmetry codes: (i) 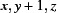; (ii) 
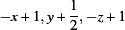
.

**Fig. 1.**
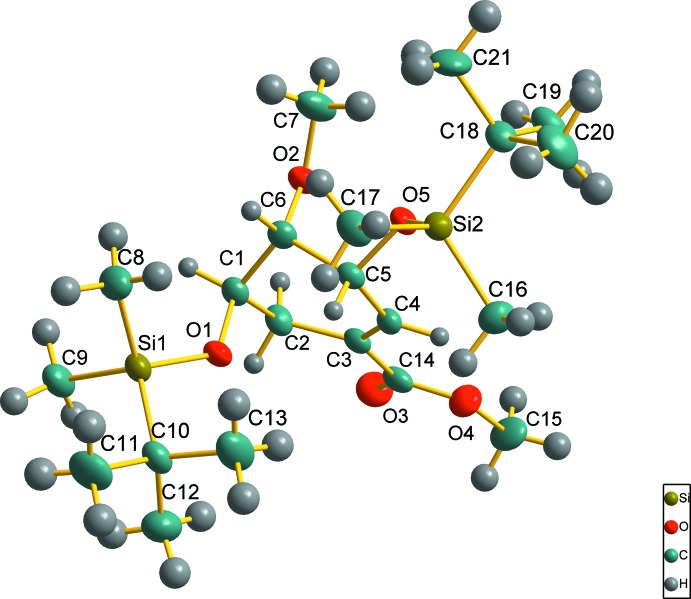
Molecular structure of C21H42O5Si2 with atom-labelling scheme and ellipsoids drawn at the 50% probability level.

## supplementary crystallographic information

### Comment

Vandetanib is a small molecule tyrosine kinase inhibitor, which can act on the tumor cells epidermal growth factor receptor (EGFR), vascular endothelial growth factor receptor (VEGFR) and the RET tyrosine kinase (Heymach, 2005; Morabito *et al.*, 2009). Vandetanib has a good therapeutic effect for Medullary thyroid cancer and Non-small cell lung cancer (Wells *et al.*, 2010; Natale *et al.*, 2009).

(3*R*,4*S*,5*R*)-Methyl 3,5-bis[(*tert*-butyldimethylsilyl)oxy]-4-methoxycyclohex-1-enecarboxylate (Fig. 1) is an intermediate to synthetize Vandetanib derivatives. Here, the synthesis and crystallographic characterization of the compound are reported.

The crystal structure of the compound has monoclinic (*P*2_1_) symmetry at 113 K. No hydrogen-bonding or π–π interactions are observed in the crystal structure. Despite the relatively large steric size of substituent groups, the cyclohexene still has a nearly ideal half-chair form with carbon atoms C3, C4,C5 and C2 lying in one plane.

### Experimental

Solid sodium hydroxide (2.8 g, 0.07 mol) was added to a stirred solution of (3R,4S,5R)-methyl 3,5-bis((*tert*-butyldimethylsilyl)oxy)-4-hydroxycyclohex-1-εnecarboxylate (5.6 g, 0.134 mol) in acetonitrile (60 ml) at room temperature.The reaction mixture was added dropwise to a solution of dimethyl sulfate (4.2 ml, 0.044 mol) in acetonitrile (60 ml). After the dropwise addition, the temperature was raised to 40 °C. The reaction was completed within 15 hrs at 40 °C stirring. The solvent was removed under reduced pressure, and ethyl acetate (300 ml) and H_2_O (100 ml) were added three times to extract the solid. The ethyl acetate layer was dried with anhydrous magnesium sulfate and a white solid was obtained after removal of the solvent. The yield was 4.8 g (82.7%). About 0.5g of the product was put in an ampoule bottle and 10 ml absolute methanol was added. The white single crystals suitable for X-ray diffraction analysis were obtained by slow evaporation of the solvent at room temperature after 1 week.

### Refinement

H atoms were placed at calculated positions with C-H = 0.98 Å (methyl), 0.99 Å (methylene), 1.00 Å (methine sp^3^) and 0.95 Å (methine sp^2^) and refined as riding atoms with *U*_iso_(H) = 1.5*U*_eq_(C) (methyl) or 1.2*U*_eq_(C) (others).

### Crystal data

**Table:**

C_21_H_42_O_5_Si_2_	*F*(000) = 472
*M**_r_* = 430.72	*D*_x_ = 1.108 Mg m^−^^3^
Monoclinic, *P*2_1_	Mo *K*α radiation, λ = 0.71073 Å
*a* = 10.760 (5) Å	Cell parameters from 4632 reflections
*b* = 8.321 (4) Å	θ = 1.9–27.9°
*c* = 14.601 (7) Å	µ = 0.16 mm^−^^1^
β = 98.997 (9)°	*T* = 113 K
*V* = 1291.3 (10) Å^3^	Prism, colourless
*Z* = 2	0.20 × 0.18 × 0.12 mm

### Data collection

**Table:**

Rigaku Saturn724 CCD diffractometer	6015 independent reflections
Radiation source: rotating anode	4456 reflections with *I* > 2σ(*I*)
Multilayer monochromator	*R*_int_ = 0.058
Detector resolution: 14.22 pixels mm^-1^	θ_max_ = 27.9°, θ_min_ = 1.9°
ω and φ scans	*h* = −14→14
Absorption correction: multi-scan (*CrystalClear*; Rigaku, 2007)	*k* = −10→10
*T*_min_ = 0.968, *T*_max_ = 0.981	*l* = −19→19
13589 measured reflections	

### Refinement

**Table:**

Refinement on *F*^2^	Secondary atom site location: difference Fourier map
Least-squares matrix: full	Hydrogen site location: inferred from neighbouring sites
*R*[*F*^2^ > 2σ(*F*^2^)] = 0.048	H-atom parameters constrained
*wR*(*F*^2^) = 0.073	*w* = 1/[σ^2^(*F*_o_^2^) + (0.010*P*)^2^] where *P* = (*F*_o_^2^ + 2*F*_c_^2^)/3
*S* = 0.98	(Δ/σ)_max_ = 0.001
6015 reflections	Δρ_max_ = 0.21 e Å^−^^3^
265 parameters	Δρ_min_ = −0.29 e Å^−^^3^
1 restraint	Absolute structure: Flack (1983), 2745 Friedel pairs
Primary atom site location: structure-invariant direct methods	Flack parameter: −0.04 (9)

### Special details

**Table:**

Geometry. All e.s.d.'s (except the e.s.d. in the dihedral angle between two l.s. planes) are estimated using the full covariance matrix. The cell e.s.d.'s are taken into account individually in the estimation of e.s.d.'s in distances, angles and torsion angles; correlations between e.s.d.'s in cell parameters are only used when they are defined by crystal symmetry. An approximate (isotropic) treatment of cell e.s.d.'s is used for estimating e.s.d.'s involving l.s. planes.
Refinement. Refinement of *F*^2^ against ALL reflections. The weighted *R*-factor *wR* and goodness of fit *S* are based on *F*^2^, conventional *R*-factors *R* are based on *F*, with *F* set to zero for negative *F*^2^. The threshold expression of *F*^2^ > σ(*F*^2^) is used only for calculating *R*-factors(gt) *etc*. and is not relevant to the choice of reflections for refinement. *R*-factors based on *F*^2^ are statistically about twice as large as those based on *F*, and *R*- factors based on ALL data will be even larger.

### Fractional atomic coordinates and isotropic or equivalent isotropic displacement parameters (Å^2^)

**Table:**

	*x*	*y*	*z*	*U*_iso_*/*U*_eq_	
Si1	0.43693 (6)	0.59832 (8)	0.75544 (5)	0.02755 (16)	
Si2	0.93837 (6)	0.92816 (8)	0.82849 (4)	0.02554 (16)	
O1	0.55081 (13)	0.59788 (19)	0.69212 (10)	0.0277 (4)	
O2	0.66743 (13)	0.92780 (19)	0.57018 (10)	0.0307 (4)	
O3	0.75863 (16)	0.3984 (2)	0.44370 (12)	0.0421 (5)	
O4	0.93371 (15)	0.4208 (2)	0.54942 (11)	0.0367 (4)	
O5	0.86351 (13)	0.89726 (17)	0.72306 (10)	0.0246 (4)	
C1	0.5623 (2)	0.7011 (3)	0.61583 (16)	0.0274 (6)	
H1	0.4777	0.7436	0.5885	0.033*	
C2	0.61777 (19)	0.6039 (3)	0.54295 (15)	0.0290 (5)	
H2A	0.5717	0.5011	0.5319	0.035*	
H2B	0.6066	0.6644	0.4838	0.035*	
C3	0.7562 (2)	0.5697 (3)	0.57327 (15)	0.0250 (5)	
C4	0.8238 (2)	0.6447 (3)	0.64413 (15)	0.0238 (5)	
H4	0.9101	0.6167	0.6598	0.029*	
C5	0.7728 (2)	0.7704 (3)	0.70088 (15)	0.0242 (5)	
H5	0.7564	0.7202	0.7601	0.029*	
C6	0.6494 (2)	0.8400 (3)	0.65060 (15)	0.0266 (6)	
H6	0.6093	0.9098	0.6935	0.032*	
C7	0.6928 (2)	1.0957 (3)	0.58602 (16)	0.0488 (7)	
H7A	0.6265	1.1434	0.6163	0.073*	
H7B	0.6950	1.1496	0.5266	0.073*	
H7C	0.7743	1.1086	0.6260	0.073*	
C8	0.4174 (2)	0.8026 (3)	0.80365 (17)	0.0441 (7)	
H8A	0.4949	0.8335	0.8444	0.066*	
H8B	0.3473	0.8016	0.8392	0.066*	
H8C	0.3998	0.8801	0.7527	0.066*	
C9	0.2855 (2)	0.5375 (3)	0.68386 (17)	0.0412 (7)	
H9A	0.2537	0.6259	0.6423	0.062*	
H9B	0.2239	0.5122	0.7246	0.062*	
H9C	0.2992	0.4426	0.6470	0.062*	
C10	0.4906 (2)	0.4488 (3)	0.84924 (16)	0.0327 (6)	
C11	0.4060 (3)	0.4598 (3)	0.92525 (17)	0.0537 (8)	
H11A	0.4329	0.3794	0.9733	0.081*	
H11B	0.3184	0.4396	0.8978	0.081*	
H11C	0.4131	0.5673	0.9529	0.081*	
C12	0.4852 (3)	0.2784 (3)	0.80998 (19)	0.0501 (8)	
H12A	0.5357	0.2727	0.7598	0.075*	
H12B	0.3977	0.2505	0.7858	0.075*	
H12C	0.5186	0.2028	0.8591	0.075*	
C13	0.6274 (2)	0.4848 (3)	0.89552 (17)	0.0515 (8)	
H13A	0.6519	0.4094	0.9467	0.077*	
H13B	0.6325	0.5950	0.9194	0.077*	
H13C	0.6842	0.4727	0.8496	0.077*	
C14	0.8129 (2)	0.4543 (3)	0.51520 (17)	0.0299 (6)	
C15	0.9983 (3)	0.3166 (3)	0.49216 (19)	0.0448 (7)	
H15A	0.9561	0.2119	0.4853	0.067*	
H15B	1.0858	0.3022	0.5216	0.067*	
H15C	0.9964	0.3656	0.4309	0.067*	
C16	1.0379 (2)	0.7499 (3)	0.86556 (17)	0.0409 (7)	
H16A	0.9843	0.6549	0.8664	0.061*	
H16B	1.0847	0.7684	0.9278	0.061*	
H16C	1.0973	0.7324	0.8220	0.061*	
C17	0.8227 (2)	0.9581 (3)	0.90962 (15)	0.0384 (7)	
H17A	0.7633	1.0430	0.8857	0.058*	
H17B	0.8674	0.9893	0.9707	0.058*	
H17C	0.7768	0.8578	0.9151	0.058*	
C18	1.0368 (2)	1.1119 (3)	0.81908 (16)	0.0314 (6)	
C19	1.1006 (2)	1.1013 (3)	0.73279 (15)	0.0429 (7)	
H19A	1.1597	1.1908	0.7326	0.064*	
H19B	1.0367	1.1069	0.6771	0.064*	
H19C	1.1462	0.9993	0.7333	0.064*	
C20	1.1383 (2)	1.1213 (4)	0.90549 (17)	0.0510 (8)	
H20A	1.1989	1.0339	0.9039	0.076*	
H20B	1.0988	1.1113	0.9613	0.076*	
H20C	1.1820	1.2248	0.9064	0.076*	
C21	0.9558 (3)	1.2647 (3)	0.8138 (2)	0.0521 (9)	
H21A	0.9137	1.2710	0.8685	0.078*	
H21B	0.8924	1.2613	0.7577	0.078*	
H21C	1.0095	1.3593	0.8116	0.078*	

### Atomic displacement parameters (Å^2^)

**Table:**

	*U*^11^	*U*^22^	*U*^33^	*U*^12^	*U*^13^	*U*^23^
Si1	0.0247 (4)	0.0286 (4)	0.0307 (4)	−0.0038 (3)	0.0084 (3)	0.0005 (3)
Si2	0.0258 (4)	0.0250 (3)	0.0258 (4)	−0.0017 (3)	0.0038 (3)	−0.0023 (3)
O1	0.0305 (9)	0.0244 (8)	0.0305 (9)	−0.0039 (8)	0.0118 (7)	0.0027 (8)
O2	0.0330 (10)	0.0296 (9)	0.0293 (9)	−0.0063 (9)	0.0039 (8)	0.0070 (8)
O3	0.0526 (12)	0.0404 (11)	0.0344 (10)	−0.0022 (10)	0.0103 (9)	−0.0126 (9)
O4	0.0402 (11)	0.0333 (9)	0.0389 (10)	0.0074 (10)	0.0137 (9)	−0.0083 (9)
O5	0.0239 (9)	0.0259 (9)	0.0238 (8)	−0.0070 (7)	0.0027 (7)	0.0000 (7)
C1	0.0271 (15)	0.0303 (13)	0.0261 (13)	−0.0032 (11)	0.0076 (12)	0.0023 (11)
C2	0.0293 (14)	0.0325 (13)	0.0261 (13)	−0.0070 (13)	0.0071 (11)	−0.0070 (13)
C3	0.0284 (13)	0.0223 (13)	0.0269 (13)	−0.0053 (10)	0.0121 (11)	−0.0011 (10)
C4	0.0228 (13)	0.0242 (13)	0.0258 (13)	−0.0020 (10)	0.0081 (11)	0.0010 (10)
C5	0.0263 (14)	0.0243 (12)	0.0228 (13)	−0.0053 (11)	0.0060 (11)	0.0002 (10)
C6	0.0297 (15)	0.0285 (13)	0.0223 (13)	−0.0015 (11)	0.0065 (11)	0.0026 (11)
C7	0.0525 (17)	0.0375 (15)	0.0521 (18)	−0.0128 (16)	−0.0055 (14)	0.0143 (16)
C8	0.0436 (18)	0.0375 (15)	0.057 (2)	0.0024 (14)	0.0250 (16)	−0.0008 (14)
C9	0.0345 (16)	0.0421 (16)	0.0460 (17)	−0.0101 (12)	0.0030 (13)	0.0066 (14)
C10	0.0306 (15)	0.0356 (15)	0.0330 (14)	−0.0086 (12)	0.0089 (12)	0.0021 (12)
C11	0.065 (2)	0.057 (2)	0.0444 (18)	−0.0087 (16)	0.0247 (16)	0.0125 (16)
C12	0.055 (2)	0.0378 (17)	0.058 (2)	−0.0007 (15)	0.0082 (17)	0.0078 (15)
C13	0.0412 (18)	0.062 (2)	0.0471 (18)	−0.0091 (15)	−0.0064 (14)	0.0198 (16)
C14	0.0361 (16)	0.0248 (14)	0.0320 (14)	−0.0073 (12)	0.0146 (12)	−0.0002 (11)
C15	0.0537 (18)	0.0387 (16)	0.0472 (17)	0.0135 (15)	0.0241 (15)	−0.0056 (14)
C16	0.0421 (17)	0.0359 (16)	0.0429 (17)	0.0046 (13)	0.0008 (14)	0.0045 (14)
C17	0.0428 (17)	0.0393 (17)	0.0353 (15)	−0.0083 (13)	0.0136 (13)	−0.0057 (13)
C18	0.0321 (14)	0.0280 (13)	0.0344 (14)	−0.0053 (13)	0.0065 (12)	−0.0077 (13)
C19	0.0434 (16)	0.0476 (15)	0.0392 (16)	−0.0200 (16)	0.0110 (13)	−0.0045 (15)
C20	0.0497 (18)	0.0577 (19)	0.0436 (18)	−0.0243 (17)	0.0011 (15)	−0.0130 (16)
C21	0.059 (2)	0.0201 (14)	0.078 (2)	−0.0042 (14)	0.0140 (19)	−0.0030 (15)

### Geometric parameters (Å, º)

**Table:**

Si1—O1	1.6464 (15)	C9—H9B	0.9800
Si1—C9	1.863 (2)	C9—H9C	0.9800
Si1—C8	1.864 (3)	C10—C12	1.527 (3)
Si1—C10	1.874 (3)	C10—C11	1.544 (3)
Si2—O5	1.6421 (16)	C10—C13	1.549 (3)
Si2—C16	1.860 (2)	C11—H11A	0.9800
Si2—C17	1.864 (2)	C11—H11B	0.9800
Si2—C18	1.877 (3)	C11—H11C	0.9800
O1—C1	1.427 (2)	C12—H12A	0.9800
O2—C6	1.422 (2)	C12—H12B	0.9800
O2—C7	1.435 (3)	C12—H12C	0.9800
O3—C14	1.207 (3)	C13—H13A	0.9800
O4—C14	1.347 (3)	C13—H13B	0.9800
O4—C15	1.454 (3)	C13—H13C	0.9800
O5—C5	1.440 (2)	C15—H15A	0.9800
C1—C6	1.523 (3)	C15—H15B	0.9800
C1—C2	1.530 (3)	C15—H15C	0.9800
C1—H1	1.0000	C16—H16A	0.9800
C2—C3	1.513 (3)	C16—H16B	0.9800
C2—H2A	0.9900	C16—H16C	0.9800
C2—H2B	0.9900	C17—H17A	0.9800
C3—C4	1.324 (3)	C17—H17B	0.9800
C3—C14	1.475 (3)	C17—H17C	0.9800
C4—C5	1.492 (3)	C18—C19	1.529 (3)
C4—H4	0.9500	C18—C20	1.536 (3)
C5—C6	1.527 (3)	C18—C21	1.537 (3)
C5—H5	1.0000	C19—H19A	0.9800
C6—H6	1.0000	C19—H19B	0.9800
C7—H7A	0.9800	C19—H19C	0.9800
C7—H7B	0.9800	C20—H20A	0.9800
C7—H7C	0.9800	C20—H20B	0.9800
C8—H8A	0.9800	C20—H20C	0.9800
C8—H8B	0.9800	C21—H21A	0.9800
C8—H8C	0.9800	C21—H21B	0.9800
C9—H9A	0.9800	C21—H21C	0.9800
			
O1—Si1—C9	110.29 (10)	C12—C10—Si1	110.67 (18)
O1—Si1—C8	110.58 (10)	C11—C10—Si1	109.69 (17)
C9—Si1—C8	108.63 (12)	C13—C10—Si1	110.70 (16)
O1—Si1—C10	103.74 (10)	C10—C11—H11A	109.5
C9—Si1—C10	111.85 (11)	C10—C11—H11B	109.5
C8—Si1—C10	111.69 (12)	H11A—C11—H11B	109.5
O5—Si2—C16	108.88 (10)	C10—C11—H11C	109.5
O5—Si2—C17	109.77 (10)	H11A—C11—H11C	109.5
C16—Si2—C17	109.45 (12)	H11B—C11—H11C	109.5
O5—Si2—C18	104.99 (10)	C10—C12—H12A	109.5
C16—Si2—C18	111.41 (12)	C10—C12—H12B	109.5
C17—Si2—C18	112.21 (11)	H12A—C12—H12B	109.5
C1—O1—Si1	126.87 (15)	C10—C12—H12C	109.5
C6—O2—C7	114.49 (18)	H12A—C12—H12C	109.5
C14—O4—C15	115.5 (2)	H12B—C12—H12C	109.5
C5—O5—Si2	122.79 (13)	C10—C13—H13A	109.5
O1—C1—C6	108.68 (18)	C10—C13—H13B	109.5
O1—C1—C2	108.40 (18)	H13A—C13—H13B	109.5
C6—C1—C2	110.27 (18)	C10—C13—H13C	109.5
O1—C1—H1	109.8	H13A—C13—H13C	109.5
C6—C1—H1	109.8	H13B—C13—H13C	109.5
C2—C1—H1	109.8	O3—C14—O4	123.3 (2)
C3—C2—C1	111.65 (19)	O3—C14—C3	124.1 (2)
C3—C2—H2A	109.3	O4—C14—C3	112.5 (2)
C1—C2—H2A	109.3	O4—C15—H15A	109.5
C3—C2—H2B	109.3	O4—C15—H15B	109.5
C1—C2—H2B	109.3	H15A—C15—H15B	109.5
H2A—C2—H2B	108.0	O4—C15—H15C	109.5
C4—C3—C14	122.0 (2)	H15A—C15—H15C	109.5
C4—C3—C2	122.4 (2)	H15B—C15—H15C	109.5
C14—C3—C2	115.5 (2)	Si2—C16—H16A	109.5
C3—C4—C5	124.0 (2)	Si2—C16—H16B	109.5
C3—C4—H4	118.0	H16A—C16—H16B	109.5
C5—C4—H4	118.0	Si2—C16—H16C	109.5
O5—C5—C4	110.08 (18)	H16A—C16—H16C	109.5
O5—C5—C6	109.77 (18)	H16B—C16—H16C	109.5
C4—C5—C6	111.52 (19)	Si2—C17—H17A	109.5
O5—C5—H5	108.5	Si2—C17—H17B	109.5
C4—C5—H5	108.5	H17A—C17—H17B	109.5
C6—C5—H5	108.5	Si2—C17—H17C	109.5
O2—C6—C1	105.72 (18)	H17A—C17—H17C	109.5
O2—C6—C5	111.78 (18)	H17B—C17—H17C	109.5
C1—C6—C5	108.40 (19)	C19—C18—C20	109.1 (2)
O2—C6—H6	110.3	C19—C18—C21	109.3 (2)
C1—C6—H6	110.3	C20—C18—C21	108.9 (2)
C5—C6—H6	110.3	C19—C18—Si2	110.14 (17)
O2—C7—H7A	109.5	C20—C18—Si2	108.56 (18)
O2—C7—H7B	109.5	C21—C18—Si2	110.82 (16)
H7A—C7—H7B	109.5	C18—C19—H19A	109.5
O2—C7—H7C	109.5	C18—C19—H19B	109.5
H7A—C7—H7C	109.5	H19A—C19—H19B	109.5
H7B—C7—H7C	109.5	C18—C19—H19C	109.5
Si1—C8—H8A	109.5	H19A—C19—H19C	109.5
Si1—C8—H8B	109.5	H19B—C19—H19C	109.5
H8A—C8—H8B	109.5	C18—C20—H20A	109.5
Si1—C8—H8C	109.5	C18—C20—H20B	109.5
H8A—C8—H8C	109.5	H20A—C20—H20B	109.5
H8B—C8—H8C	109.5	C18—C20—H20C	109.5
Si1—C9—H9A	109.5	H20A—C20—H20C	109.5
Si1—C9—H9B	109.5	H20B—C20—H20C	109.5
H9A—C9—H9B	109.5	C18—C21—H21A	109.5
Si1—C9—H9C	109.5	C18—C21—H21B	109.5
H9A—C9—H9C	109.5	H21A—C21—H21B	109.5
H9B—C9—H9C	109.5	C18—C21—H21C	109.5
C12—C10—C11	109.4 (2)	H21A—C21—H21C	109.5
C12—C10—C13	108.7 (2)	H21B—C21—H21C	109.5
C11—C10—C13	107.5 (2)		
			
C9—Si1—O1—C1	65.82 (19)	O5—C5—C6—C1	171.87 (16)
C8—Si1—O1—C1	−54.4 (2)	C4—C5—C6—C1	49.6 (2)
C10—Si1—O1—C1	−174.24 (17)	O1—Si1—C10—C12	−70.14 (19)
C16—Si2—O5—C5	63.48 (18)	C9—Si1—C10—C12	48.7 (2)
C17—Si2—O5—C5	−56.31 (18)	C8—Si1—C10—C12	170.72 (18)
C18—Si2—O5—C5	−177.12 (16)	O1—Si1—C10—C11	169.01 (16)
Si1—O1—C1—C6	97.0 (2)	C9—Si1—C10—C11	−72.1 (2)
Si1—O1—C1—C2	−143.19 (15)	C8—Si1—C10—C11	49.9 (2)
O1—C1—C2—C3	−73.1 (2)	O1—Si1—C10—C13	50.48 (19)
C6—C1—C2—C3	45.8 (3)	C9—Si1—C10—C13	169.35 (17)
C1—C2—C3—C4	−13.4 (3)	C8—Si1—C10—C13	−68.6 (2)
C1—C2—C3—C14	170.15 (18)	C15—O4—C14—O3	2.6 (3)
C14—C3—C4—C5	175.70 (19)	C15—O4—C14—C3	−175.76 (18)
C2—C3—C4—C5	−0.5 (3)	C4—C3—C14—O3	−170.6 (2)
Si2—O5—C5—C4	−110.40 (18)	C2—C3—C14—O3	5.9 (3)
Si2—O5—C5—C6	126.48 (16)	C4—C3—C14—O4	7.8 (3)
C3—C4—C5—O5	−140.3 (2)	C2—C3—C14—O4	−175.7 (2)
C3—C4—C5—C6	−18.3 (3)	O5—Si2—C18—C19	−43.92 (18)
C7—O2—C6—C1	151.68 (18)	C16—Si2—C18—C19	73.8 (2)
C7—O2—C6—C5	−90.6 (2)	C17—Si2—C18—C19	−163.11 (17)
O1—C1—C6—O2	173.92 (17)	O5—Si2—C18—C20	−163.30 (16)
C2—C1—C6—O2	55.2 (2)	C16—Si2—C18—C20	−45.6 (2)
O1—C1—C6—C5	53.9 (2)	C17—Si2—C18—C20	77.52 (19)
C2—C1—C6—C5	−64.8 (2)	O5—Si2—C18—C21	77.13 (18)
O5—C5—C6—O2	55.7 (2)	C16—Si2—C18—C21	−165.17 (17)
C4—C5—C6—O2	−66.5 (2)	C17—Si2—C18—C21	−42.1 (2)

### Hydrogen-bond geometry (Å, º)

**Table:**

*D*—H···*A*	*D*—H	H···*A*	*D*···*A*	*D*—H···*A*
C7—H7*B*···O3^i^	0.98	2.55	3.410 (3)	147
C9—H9*A*···O3^ii^	0.98	2.59	3.527 (3)	161

Symmetry codes: (i) *x*, *y*+1, *z*; (ii) −*x*+1, *y*+1/2, −*z*+1.
